# Using machine learning methods to predict in-hospital mortality of sepsis patients in the ICU

**DOI:** 10.1186/s12911-020-01271-2

**Published:** 2020-10-02

**Authors:** Guilan Kong, Ke Lin, Yonghua Hu

**Affiliations:** 1 National Institute of Health Data Science, Peking University, Beijing, China; 2 Center for Data Science in Health and Medicine, Peking University, Beijing, China; 3 Department of Epidemiology and Biostatistics, School of Public Health, Peking University, Beijing, China; 4 Medical Informatics Center, Peking University, Beijing, China

**Keywords:** Intensive care unit, Sepsis, Prediction model, Machine learning, In-hospital mortality

## Abstract

**Background:**

Early and accurate identification of sepsis patients with high risk of in-hospital death can help physicians in intensive care units (ICUs) make optimal clinical decisions. This study aimed to develop machine learning-based tools to predict the risk of hospital death of patients with sepsis in ICUs.

**Methods:**

The source database used for model development and validation is the medical information mart for intensive care (MIMIC) III. We identified adult sepsis patients using the new sepsis definition Sepsis-3. A total of 86 predictor variables consisting of demographics, laboratory tests and comorbidities were used. We employed the least absolute shrinkage and selection operator (LASSO), random forest (RF), gradient boosting machine (GBM) and the traditional logistic regression (LR) method to develop prediction models. In addition, the prediction performance of the four developed models was evaluated and compared with that of an existent scoring tool – simplified acute physiology score (SAPS) II – using five different performance measures: the area under the receiver operating characteristic curve (AUROC), Brier score, sensitivity, specificity and calibration plot.

**Results:**

The records of 16,688 sepsis patients in MIMIC III were used for model training and test. Amongst them, 2949 (17.7%) patients had in-hospital death. The average AUROCs of the LASSO, RF, GBM, LR and SAPS II models were 0.829, 0.829, 0.845, 0.833 and 0.77, respectively. The Brier scores of the LASSO, RF, GBM, LR and SAPS II models were 0.108, 0.109, 0.104, 0.107 and 0.146, respectively. The calibration plots showed that the GBM, LASSO and LR models had good calibration; the RF model underestimated high-risk patients; and SAPS II had the poorest calibration.

**Conclusion:**

The machine learning-based models developed in this study had good prediction performance. Amongst them, the GBM model showed the best performance in predicting the risk of in-hospital death. It has the potential to assist physicians in the ICU to perform appropriate clinical interventions for critically ill sepsis patients and thus may help improve the prognoses of sepsis patients in the ICU.

## Background

Sepsis is a life-threatening illness that occurs when the body’s response to infection is out of balance [1]. It can trigger body changes that may damage multiple organ systems and lead to death [2]. Sepsis has become a major contributor of public health and economic burden [3, 4]. It is associated with high risk of complications and in-hospital death, longer hospital stays and higher medical costs. Today, sepsis has become a major cause of in-hospital death for intensive care unit (ICU) patients. In the US, 10% of patients admitted to the ICU have sepsis, and around 25% of ICU beds are occupied by sepsis patients [5–7]. Given the high mortality of sepsis patients in the ICU, the risk of in-hospital death of patients with sepsis needs to be discovered the earlier the better. Early and accurate identification of sepsis patients with high risk of in-hospital death can help ICU physicians make optimal clinical decisions, which can, in turn, improve their clinical outcomes [8].

The initial sepsis definition was proposed in 1991, referred as Sepsis-1 [9]. It was defined as infected patients meeting two or more of the systemic inflammatory response syndrome (SIRS) criteria, including 1) temperature > 38 °C or < 36 °C, 2) heart rate > 90/min, 3) respiratory rate > 20/min or partial pressure of carbon dioxide (PaCO_2_) < 32 mmHg (4.3 kPa), and 4) white blood cell count (WBC) > 12,000/mm^3^ or < 4000/mm^3^ or > 10% immature (band) forms. Sepsis-1 was updated to Sepsis-2 [10] in 2001 by expanding the list of diagnostic criteria but did not offer alternatives. In effect, the definition of sepsis has remained largely unchanged for more than two decades. In 2016, a new sepsis definition, Sepsis-3 [1], was proposed. In Sepsis-3, instead of checking the SIRS criteria as in previous definitions, infected patients who have a sequential organ failure assessment (SOFA) score [11] higher than 2 are defined as having sepsis.

Some scoring tools have been developed to assess the illness severity of patients with sepsis. Amongst them, the simplified acute physiology score (SAPS) II [12]; the acute physiology and chronic health evaluation (APACHE) II, III and IV scores [13–15]; and the SOFA score are frequently used severity assessment tools in the ICU. Patient vital signs, laboratory results and demographic statistics are risk factors used in these scoring systems for severity assessment. Most of these severity scores were developed decades ago. They may perform well in the population or clinical settings at the time when they were developed. However, the performance of these severity scores has declined due to the fact that the population and clinical settings have changed over the time. Moreover, some studies [16, 17] showed that the calibration and discrimination capabilities of these severity scores are poor in predicting the risk of in-hospital death of sepsis patients. In addition, there is a specific severity score particularly for sepsis patients: Sepsis Severity Score (SSS) [18]. The SSS was developed as a specific severity score for predicting in-hospital mortality of sepsis patients. However, a study [17] showed that the discrimination performance of SSS is not as good as that of APACHE IV in predicting the risk of in-hospital death of patients with sepsis, and the calibration capability of SSS is poor.

Given the poor performance of existing severity scores, some new models have been developed for predicting the risk of in-hospital death amongst ICU patients with sepsis [19–21]. Fang et al. [22] developed and validated a scoring system for predicting 28-day mortality risk of patients with sepsis. The corresponding area under the receiver operating characteristic curve (AUROC) generated by the scoring system is 0.789. Xie et al. [23] used clinical features and biomarkers as predictors to develop a model based on traditional logistic regression (LR) algorithm for predicting the mortality risk of sepsis patients. The AUROC of their model is 0.778.

With the accumulation of big data and the development of techniques for data storage, machine learning methods have attracted considerable research attention [24–26]. Several innovative and pragmatic machine learning methods such as random forest (RF) [27], gradient boosting machine (GBM) [28] and the least absolute shrinkage and selection operator (LASSO) [29] which is a type of linear regression using shrinkage, have been proposed, and these models have good prediction performance in medicine. Some machine learning-based models have been developed for ICU mortality prediction in the literature [30, 31]. In sepsis area, Zhang et al. [32] used the LASSO method to build a tool for predicting the mortality risk of sepsis patients based on the medical information mart for intensive care (MIMIC) III dataset [33], and their research results showed that the LASSO-based prediction model was superior to SOFA score in discrimination. Taylor et al. [34] employed machine learning methods to build mortality prediction models for patients with sepsis, and their research results showed that the RF model performed better than the LR model in discrimination. Relevant studies about mortality prediction for sepsis patients are listed in Table 1, where the dataset, methodology, predictors, outcome and sepsis definition used in each study were presented.

**Table 1:** Relevant studies about mortality prediction for sepsis patients

Authors	Title	Dataset	Methodology	Predictors	Outcome	Sepsis definition
Masson, S. et al. [19]	Presepsin (soluble CD14 subtype) and procalcitonin levels for mortality prediction in sepsis: data from the Albumin Italian Outcome Sepsis trial	A multicentre, randomised Albumin Italian Outcome Sepsis trial, 100 patients	Cox regression model	Presepsin level, procalcitonin level and some covariates	28-day/ICU/90-day mortality	Sepsis-2
Adrie C. et al. [20]	Model for predicting short-term mortality of severe sepsis	A multicentre database including data from 12 ICUs, 2268 patients	Generalised linear model	SAPS II and LOD scores at ICU admission, septic shock, multiple organ failure, comorbidities, procedures, agents, bacteraemia and sources of infection	14-day mortality within ICU stay	Sepsis-2
Ripoll, V.J.R. et al. [21]	Sepsis mortality prediction with the Quotient Basis Kernel	MIMIC II	Support vector machines (SVMs), LR, SAPS	SOFA and SAPS scores at ICU admission	ICU mortality	Sepsis-2
Fang W-F et al. [22]	Development and validation of immune dysfunction score to predict 28-day mortality of sepsis patients	Sepsis patients admitted to ICU at a hospital in Taiwan, 151 patients	LR	Monocyte HLA-DR^*^ expression, plasma G-CSF^*^ level, plasma IL^*^-10 level, and serum SeMo^*^ ratio	28-day mortality	Sepsis-3
Xie, Y. et al. [23]	Using clinical features and biomarkers to predict 60-day mortality of sepsis patients	Protocol-based care in early septic shock trial, around 530 patients	LR	Clinical features and biomarkers obtained during the first 24 h of hospital admission	60-day mortality	Not mentioned
Poucke, S.V. et al. [31]	Scalable predictive analysis in critically ill patients using a visual open data analysis platform	MIMIC II	Naïve Bayes, LR, RF, AdaBoost, Bagging, Stacking, SVM	Demographics, comorbidities, types of care unit, platelet count	ICU mortality	NA
Zhang, Z. & Hong, Y [32].	Development of a novel score for the prediction of hospital mortality in patients with severe sepsis: the use of electronic healthcare records with LASSO regression	MIMIC III	LASSO, LR	Demographics, clinical and laboratory variables recorded during the first 24 h in ICU	Hospital mortality	Sepsis-2
Taylor, R.A. et al. [34]	Prediction of in-hospital mortality in emergency department patients with sepsis: a local big data-driven, machine learning approach	Adult ED^*^ visits over 12 months, 4676 patients	RF, CART, LR	Demographics, previous health status, ED health status, ED services rendered and operational details	Hospital mortality	Sepsis-2
Pregernig, A. et al. [35]	Prediction of mortality in adult patients with sepsis using six biomarkers: a systematic review and meta-analysis	44 articles in English	Qualitative analysis, meta-analysis	Angiopoietin 1 and 2, HMGB1^*^, sRAGE^*^, sTREM^*^-1, suPAR^*^	28-day/30-day/ICU/hospital/90-day mortality	Sepsis-1/Sepsis-2/Sepsis-3

^*^Abbreviations: *HLA-DR* Human leukocyte antigen D-related, ^*^G-CSF Granulocyte-colony stimulating factor, *IL* Interleukin, *SeMo* Segmented neutrophil-to-monocyte, *ED* Emergency department, *HMGB1* High mobility group box 1 protein, *sRAGE* soluble receptor for advanced glycation endproducts, *sTREM* soluble triggering receptor expressed on myeloid cells 1, *suPAR* soluble urokinase-type plasminogen activator receptor

From Table 1, we can find that most of the existing relevant studies have limitations such as limited sample sizes, limited predictor variables and old sepsis definitions, and most of them used traditional analytic methods such as LR to build models. LR models assume that the dependent variable has a linear functional relationship with predictor variables after a logit transformation, and this assumption may affect the model’s discrimination power as the relationship may be non-linear [36]. A previous study [37] recommended using the GBM model to predict the in-hospital mortality for sepsis as it often outperforms the RF model. However, few studies have employed the GBM model to predict the mortality of ICU sepsis patients.

Therefore, driven by the need of using big data and the latest sepsis definition to develop in-hospital mortality prediction models for sepsis patients in the ICU, we used the GBM, RF, LASSO and LR methods together with the latest sepsis definition criteria to build models for predicting the risk of in-hospital death of patients with sepsis in the ICU and compared their prediction performance with the existent scoring tool SAPS II.

## Methods

### Dataset and subjects

MIMIC III, an ICU database from the Beth Israel Deaconess Medical Center (BIDMC) [33], was employed for model derivation and validation. As a database accessible to researchers worldwide, MIMIC III contains over 40,000 records of patients receiving critical care in the ICU at BIDMC between 2001 and 2012. The diagnostic codes, vital signs, laboratory tests, demographics and some other clinical characteristics of each patient were included in MIMIC III. The Institutional Review Board of the BIDMC and Massachusetts Institute of Technology approved the research use of MIMIC III for researchers having attended their training course.

Adult patients between 18 and 90 years old were included in this study. We identified adult sepsis patients using the latest Sepsis-3. According to the Sepsis-3, infected patients who have a SOFA score higher than 2 are defined as having sepsis. First of all, we need to identify infected patients. We employed the International Classification of Diseases, ninth revision, Clinical Modification (ICD-9-CM) diagnosis codes provided by Angus et al. [38] to identify infected patients from the MIMIC-III database. Furthermore, we used the SOFA score as another criterion to identify sepsis patients from the infected. For those patients with twice or more ICU admissions during one hospitalisation, we included only the patient’s first ICU admission. Patients whose records have a predictor variable missing rate higher than 30% were excluded.

### Predictor variables and the primary outcome

The primary outcome of this study is in-hospital mortality of sepsis patients in the ICU.

In the process of predictor selection, we made reference to the established scoring tools, SAPS II and APACHE III, and considered variables such as obesity, serum lactate and international normalised ratio (INR), which have been found relevant to mortality, as predictors to construct the mortality prediction models [32, 38].

To ensure the availability of all predictor variables in prediction model development, we excluded variables with data missing rate higher than 30%. Finally, a total of 86 predictor variables consisting of demographics, laboratory tests and comorbidities were used as independent predictor variables for model development.

MIMIC-III has some time-stamped physiological data. For example, blood pressure and heart rate are measured hourly. In model construction, as variations and sudden changes could be more informative than average values of time-stamped measurements, the minimum and maximum values during the first 24 h of these variables for each ICU stay were used as parallel inputs for prediction models. All predictor variables are listed in Table 2. All predictor variables were extracted or calculated from the patient data recorded in the MIMIC III database.

**Table 2:** Predictor variables used in this study

Predictors	
**Acute physiology (first 24 h in the ICU)**	**Chronic health status**
Heart rate*	Elixhauser comorbidity index
Systolic blood pressure*	Congestive heart failure
Diastolic blood pressure*	Cardiac arrhythmias
Mean blood pressure*	Valvular heart disease
Respiratory rate*	Pulmonary circulation
Temperature*	Peripheral vascular
SpO2* (blood oxygen saturation)	Hypertension
Total CO_2_*	Other neurological diseases
pCO_2_* (partial pressure of CO_2_)	Chronic obstructive pulmonary disease
pH* (acidity in the blood)	Diabetes without complications
Urine output	Diabetes with complications
Glasgow Coma Score (GCS)	Hypothyroidism
GCS (eye)	Renal failure
GCS (motor)	Liver disease
GCS (verbal)	Metastatic cancer
Anion gap*	Coagulopathy
Bicarbonate*	Obesity
Creatinine*	Fluid electrolyte
Chloride*	Alcohol abuse
Glucose*	Depression
Haematocrit*	Renal replacement therapy
Haemoglobin*	**Other**
Lactate*	Gender
Platelet*	Weight loss
Potassium*	Ventilation
Partial thromboplastin time*	Age
INR*	Weight
Prothrombin time*	SAPS II score (first 24 h in the ICU)
Sodium*	SOFA score (first 24 h in the ICU)
Blood urea nitrogen (BUN)*	
WBC*	
Acute kidney injury	

*: each predictor marked with * means that it is a time-stamped variable, and its corresponding minimum and maximum values within the first 24 h in the ICU were used as inputs in model development

### Prediction models

In this study, three machine learning methods, namely, LASSO, RF and GBM, together with the frequently used LR method were used to develop in-hospital mortality prediction models. In addition, the established scoring tool, SAPS II, was employed for the prediction. A brief introduction of the SAPS II, LR, LASSO, RF and GBM models is provided as follows.

#### SAPS II

SAPS II is a scoring tool developed to measure the disease severity of patients (≥15) admitted to ICUs. SAPS II was developed based on a large international sample of patients. It can provide an estimate of the risk of death without having to specify a primary diagnosis. Detailed variables and score assignment in SAPS II are shown in Table 3.

**Table 3:** Variables and score assignment in SAPS II

Variables		Maximum scores
Acute physiology	Temperature	3
	Heart rate	11
	Systolic blood pressure	13
	WBC	12
	Bilirubin	9
	Serum sodium	5
	Serum potassium	3
	Serum bicarbonate	6
	BUN	10
	Urine output	11
	PaO_2_^a^or FiO_2_^a^	11
	GCS	26
Chronic health status	AIDS^a^	17
	Haematologic malignancy	10
	Metastatic cancer	9
Other	Age	18
	Type of admission	8
Overall score		182

^a^*Abbreviations*: *AIDS* Acquired immunodeficiency syndrome, *PaO*_*2*_ Partial pressure of oxygen, *FiO*_*2*_ Fraction of inspired oxygen

The probability of in-hospital death is estimated from the SAPS II score as follows:

1 $$ \mathrm{logit}=-7.763+\left(0.0737+\mathrm{SAPS}\ \mathrm{II}\ \mathrm{score}\right)+\Big(0.9971\times \ln \left(\mathrm{SAPS}\ \mathrm{II}\ \mathrm{score}+1\right) $$

2 $$ p=\frac{e^{\mathrm{logit}}}{1+{e}^{\mathrm{logit}}} $$

### LR

The LR method has been widely used in medical research. The mathematical function between predictors and the risk of in-hospital death can be described as follows:

3 $$ \mathrm{logit}=\mathit{\log}\left(\frac{p}{1-p}\right)={\beta}_0+{\beta}_1{x}_1+{\beta}_2{x}_2+\cdots +{\beta}_i{x}_i+\cdots +{\beta}_N{x}_N $$

where *p* denotes the probability of in-hospital death, *x*_*i*_(*i* = 1, 2, …, *N*) represents independent predictors, *β*_*i*_(*i* = 1, 2, …, *N*) are the coefficients associated with predictors, and *N* is the number of all predictors.

#### LASSO

The LASSO model was developed in 1996. It is similar to linear regression except that it shrinks the coefficients of some variables with multicollinearity towards zero via regularisation [39]. As a result, the coefficients of some correlated predictors will be zero after model training, and a so-called sparse model with only important predictors can be generated.

In LASSO regression, the relationship between all potential predictor variables *x*_*ij*_(*i* = 1, 2, …, *N*; *j* = 1, 2, …, *P*) and the outcome *y*_*i*_(*i* = 1, 2, …, *N*) is assumed to be similar to that in linear regression. Here, *N* denotes the number of all cases in the training dataset, and *P* denotes the number of all predictor variables. Furthermore, the LASSO regression is different from linear regression in that it uses coefficient shrinkage. The coefficients in the LASSO regression are generated through model training by solving the following objective function:

4 $$ \underset{\beta }{\min}\left\{{\sum}_{i=1}^N{\left({y}_i-{\beta}_0-{\sum}_{j=1}^P{x}_{ij}{\beta}_j\right)}^2+\lambda {\sum}_{j=1}^P\left|{\beta}_j\right|\right\} $$

The first half of the objective function is about the training loss, which is the difference between model generated results and the observed outcomes, measuring how well the model fits the training dataset. The second half of the objective function is for regularisation, the penalty on the coefficients, which measures the complexity of the model. It is through the regularisation that coefficients of correlated variables can be shrinked to 0 in LASSO regression. In the function, the parameter *λ* ≥ 0 is for controlling the amount of shrinkage, and a larger *λ* means a greater shrinkage amount.

We used the glmnet package in R (https://www.r-project.org) software to train the LASSO model. In the LASSO model training, the penalty on the *β*-coefficients is controlled by the tuning parameter *λ*, and the optimal *λ* was found via cross-validation (CV) in the glmnet package.

#### RF

The RF model is a type of ensemble-learning model that uses multiple decision trees as its base models, and a majority voting system is used as the final aggregation method to synthesise the classification results of all the base models [27]. The training of decision trees in a RF model utilises the same learning algorithm, which uses Gini index [40] as the criterion for selecting appropriate variables for different nodes in tree growing. For more detailed introduction of the RF model, readers can refer to article [41].

We used the randomForest package in R to fit the RF model. In the RF model training, we set the total number of variables in each decision tree as the default value in R. We set the candidate numbers of all decision trees in the RF model to 500, 1000, 1500 and 2000, and the number that resulted in the highest AUROC was selected. We finally selected 1000 as the number of decision trees in our RF model.

#### GBM

While the RF model is an ensemble of parallel decision trees, the GBM model builds an ensemble of decision trees in a sequential way, in which the training purpose of each decision tree is to minimise the discrepancies between the observed and predicted outcomes made by all its preceding tress [28, 42]. In a GBM model, the ensemble of decision trees is trained sequentially, where each decision tree gradually corrects for the residuals of all its preceding trees via the gradient descent method. The training procedure of a GBM model consecutively and iteratively fits new decision trees to generate estimates closer to the observed outcomes. The training of the GBM model is illustrated as follows.

Let *M* be the number of all iterations or the number of all decision trees in a GBM model. *F*_*m* − 1_(*x*)(*m* = 2, 3…, *M*) is used to represent the GBM model in current status, which contains *m-*1 decision trees. Then, *h*_*m*_(*x*)(*m* = 1, 2, …, *M*) is used to represent each decision tree that the GBM model attempts to find and include. Each single decision tree *h*_*m*_(*x*) is trained using the gradient boosting method, where the negative gradient of the loss function defined for the GBM model is used for model fitting. *F*_*m*_(*x*) is used to represent the GBM model after including the *m*th decision tree *h*_*m*_(*x*). The iterative updating process of the GBM model, *F*_*m*_(*x*), can be written as follows:

5 $$ {F}_m(x)={F}_{m-1}(x)+\upnu {\gamma}_m{h}_m(x) $$

In the above formula, the coefficient *γ*_*m*_ is calculated in the process of minimising the loss function of *F*_*m*_(*x*) by a line search strategy. Overfitting is avoided by using shrinkage as a regularisation method, where *h*_*m*_(*x*) is multiplied by a small learning rate ν in each training iteration. *F*_*M*_(*x*) is the final model in which *m* reaches *M*.

We used the gbm package in R software to fit the GBM model. The grid search, an exhaustive searching strategy which attempts to find optimal parameters through a subset of manually specified values, was employed in the GBM model training. We set the candidate numbers of decision trees in the ensemble GBM model to 500, 1000, 1500 and 2000 and the candidate learning rates to 0.01 and 0.001. These candidate values were selected in accordance with a previous study by Hastie et al. [43]. We finally selected 2000 as the number of trees in the ensemble model and 0.01 as the learning rate.

The hypothesis of this study was that machine learning-based prediction models perform better than traditional regression models and existing scoring tools, and ensemble-learning models perform better than single-learning models.

### Statistical analysis

After patient and variable exclusion, the mean value of each variable was utilised to compensate for the missing values of the corresponding variable for final analysis. We employed R 3.40 to perform calculations and statistical analysis. We employed mean ± standard deviation, percentages or actual numbers to describe patient data characteristics. We employed five-fold CV to find optimal model parameters and the model with best prediction performance amongst the developed models. By using the five-fold CV method, the whole dataset was divided into five folds, and five rounds of model training and test were conducted. In each round, four folds were used as the training dataset, and the last one was used as the test dataset. The test fold was different in each round. In this study, the model with the best average prediction performance on five test datasets was considered the optimal model.

We compared the developed models using five different performance measures: Brier score [44], AUROC [45], sensitivity, specificity and calibration plot. Brier score measures the overall prediction performance, which denotes the accuracy of a prediction, of a model. The best possible score is 0 for a totally accurate prediction, whereas the lowest possible score is 1 for a wholly inaccurate prediction. AUROC is an overall measure of discrimination of a model considering sensitivity and specificity, and it denotes the capability to distinguish the survivors from the deceased. Calibration plot is an approach to illustrate the calibration capability of a model, representing the consistency between the predictions and observed outcomes.

## Results

A total of 16,688 patients with sepsis were included in model derivation, amongst which 2949 (17.7%) died. The proportions of males in all patients, in survivors and in decedents were 54.5, 54.1 and 56.05%, respectively. The average age of all patients, survivors and decedents were 65.61+/− 15.01, 65.00+/− 15.11 and 68.46+/− 14.19 years old, respectively. The average SOFA scores in all patients, the survivors and decedents were 5.57+/− 3.06, 5.10+/− 2.67 and 7.77+/− 3.72, respectively. Table 4 describes the characteristics of sepsis patients included in this study. Compared with the survivors, the decedents were older and had higher SOFA and SAPS II scores, longer ICU stays and shorter hospitals stays.

**Table 4:** Characteristics of included sepsis patients

Items	Statistics		
	All	Survivors	Decedents
Total number	16,688	13,739	2949
Age (year)	65.61 ± 15.01	65.00 ± 15.11	68.46 ± 14.19
Gender			
Male	9087 (54.5%)	7434 (54.1%)	1653 (56.05%)
Female	7601 (45.5%)	6305 (45.9%)	1296 (43.95%)
SOFA score, 1st day of ICU admission	5.57 ± 3.06	5.10 ± 2.67	7.77 ± 3.72
SAPS II score, 1st day of ICU admission	40.87 ± 13.51	38.55 ± 12.18	51.68 ± 14.13
Hospital length of stay (day)	14.42 ± 14.77	14.55 ± 14.26	13.82 ± 16.96
ICU length of stay (day)	6.41 ± 8.52	6.07 ± 8.30	7.99 ± 9.34
Number of deaths	2949 (17.7%)	NA	NA

Table 5 presents the average AUROC value, Brier score, the sensitivity and specificity corresponding to the optimal cut-off point of each model in fivefold CV. The average AUROC values of the LASSO, GBM, RF, LR and SAPS II models were 0.829 (95% confidence interval (CI): 0.827–0.831), 0.845 (95%CI: 0.837–0.853), 0.829 (95%CI: 0.823–0.834), 0.833 (95%CI: 0.830–0.838) and 0.77 (95%CI: 0.760–0.780), respectively. The average Brier scores of the LASSO, GBM, RF, LR and SAPS II models were 0.108 (95%CI: 0.107–0.109), 0.104 (95%CI: 0.102–0.105), 0.109 (95%CI: 0.108–0.109), 0.107 (95%CI: 0.105–0.108) and 0.146 (95%CI: 0.142–0.150), respectively.

**Table 5:** Performance comparison of five models

Model	Overall performance		Discrimination					
	Brier score	95% CI	AUROC	95% CI	Sensitivity	95% CI	Specificity	95% CI
LASSO	0.108	0.107–0.109	0.829	0.827–0.831	0.744	0.721–0.767	0.754	0.731–0.777
GBM	0.104	0.102–0.105	0.845	0.837–0.853	0.771	0.750–0.792	0.755	0.722–0.789
RF	0.109	0.108–0.109	0.829	0.823–0.834	0.765	0.756–0.774	0.740	0.719–0.761
LR	0.107	0.105–0.108	0.833	0.830–0.838	0.760	0.740–0.780	0.748	0.724–0.772
SAPS II	0.146	0.142–0.150	0.77	0.760–0.780	0.697	0.668–0.725	0.714	0.695–0.734

**Fig. 1:**
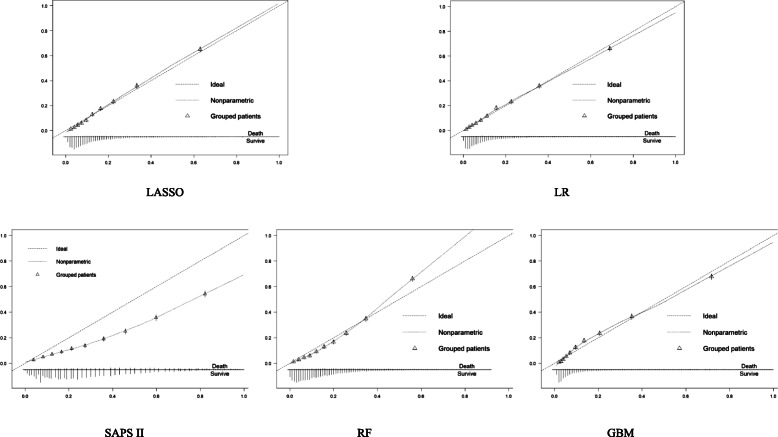
Calibration plots of the LASSO, LR, SAPS II, RF and GBM models

Figure 1 illustrates the calibration plots of the five models. It shows that the LASSO, LR and GBM models had good calibration, whereas the RF model underestimated the mortality risk of high-risk sepsis patients. Meanwhile, the SAPS II model showed the poorest calibration.

## Discussion

In this study, sepsis patient data were first extracted from the MIMIC III database. Then, four data-based tools, including LASSO, GBM, RF and LR models, were developed to perform in-hospital mortality prediction. Finally, the prediction performances of the fours tools were compared with the existent scoring tool SAPS II.

In general, the criteria for modelling method selection include model performance and interpretability. As for the model performance, ensemble-learning models usually have better prediction performance than single-learning models [46]. Regarding model interpretability, traditional regression models have considerably better interpretability than black-box machine learning models. Therefore, we considered machine learning and regression models and ensemble- and single-learning models in model selection. Finally, we selected the LASSO, GBM, RF and LR models. As an ensemble model, the RF model has good prediction performance and good interpretability. The GBM model is also an ensemble model with good performance, but its interpretability is poorer than that of the RF model. The LASSO model is a type of regression model with good interpretability but fair performance, whereas the LR model is a traditional regression model with good interpretability. We attempted to find a trade-off between the model performance and interpretability in model selection.

Regarding identification of sepsis patients using Sepsis-3, a SOFA score can be calculated for each patient in MIMIC III database and assessed whether the number is larger than 2. However, as infections have varied causes and infected sites, different diagnoses may be provided for infected patients. Thus, various diseases are related to infections. In this study, we employed the ICD codes provided by Angus et al. [38] to identify infected patients from MIMIC III. These ICD codes for infections have already been tested by a study [47]. Therefore, ICD codes can be reasonably used to identify sepsis patients from MIMIC III.

In this study, the observed mortality rate of the whole dataset was around 17.7%. However, we performed no processing on the imbalance because the prediction performance of all models developed from the dataset were acceptable. To eliminate the effect caused by data imbalance on the trained models, we employed the AUROC, which considers sensitivity and specificity and cannot be wavered by data imbalance, to evaluate prediction performance. Therefore, data imbalance would not affect the identification of optimal prediction models.

As for the predictor variable inclusion, as this study aimed to develop in-hospital mortality prediction models based on all available physiology, chronic health status, demographics and several other hospitalisation-related variables during the first 24 h after ICU admission, we excluded medications or procedures, which would alter the course of health trajectory of sepsis patients. All predictor variables used in this study aimed for mortality prediction instead of sepsis diagnosis. Thus, these predictors may include both pre-sepsis and post-sepsis variables but are restricted to the first 24 h after ICU admission. Similar to the established scoring tools SAPS II and APACHE III, the models developed in this study are stationary and can be used after the first 24 h.

Amongst the five prediction models, the GBM model had the best discrimination and overall performance, and it also had a good calibration as illustrated by the calibration plot. Unlike the LASSO and LR models, the RF and GBM models are ensemble learning models with decision tree as base models; both belong to a type of non-parametric machine learning technique having no requirements of distribution or parameter of the training dataset [48]. Ensemble learning methods based on decision tress are superior to learning techniques with parametric requirements, such as LR, because non-parametric methods have advantages in handling high-volume data without specific distribution patterns. Although the basic idea behind the GBM and RF models is to aggregate many individual weaker decision trees into an ensemble and stronger learner, a GBM model generally produces better performance than a RF model [42, 49, 50]. A RF model trains each tree independently and uses a random sample of the data for training, whereas a GBM model builds an ensemble of decision trees in a sequential way, in which each new decision tree is fitted through correcting for the residuals of all its preceding trees. This condition means that each new tree in a GBM model corrects the errors made by all previously trained trees in the model [42]. However, the GBM model has poorer interpretability than RF. The LR model performed worse than the GBM model by all measures but showed better performance than the RF and LASSO models in terms of Brier score and AUROC. The SAPS II model exhibited the poorest prediction performance amongst all these models. This condition implies that the SAPS II model requires customisation when applied to a different patient population.

In effect, machine learning-based prediction models have advantages in handling high-dimension data, which indicates that more clinical variables can be considered as model inputs than those used in existing severity scoring systems, with the benefit of discovering meaningful clinical variables that have prediction effects on in-hospital mortality.

Compared with existing studies, our research has several strengths. Firstly, we extracted sepsis patient data from the MIMIC-III dataset using the latest Sepsis-3. Secondly, we used three different machine learning methods LASSO, RF and GBM for prediction model development and compared their prediction performances with those of traditional LR and SAPS II models. The originality of this study lies in clinical application other than methodology. For the first time in the literature, the GBM model was compared with RF, LASSO, LR and SAPS II models in developing in-hospital mortality prediction models for sepsis patient in ICU.

This study also has limitations. Firstly, the MIMIC-III dataset is a dataset containing only patients from a single medical centre. The application of the developed GBM-based model to other datasets or population needs further clinical evaluation. Secondly, the data used for prediction model training and test was a subset of MIMIC-III dataset. The datasets regarding high-resolution waveforms were excluded in the analysis in this study. Thirdly, the developed models were based on the baseline data during the first 24 h in ICU and cannot be used to provide dynamic prediction.

## Conclusions

This study contributes to clinical areas with an optimal GBM-based in-hospital mortality prediction model for sepsis patients in ICU. The prediction model has the potential to aid ICU physicians to determine which patients have a high mortality risk and who should be prioritised in treatment, thus enabling them to make optimal clinical interventions and improve prognoses of sepsis patients. As all the variables used in this study for model construction are routinely collected by the information systems in ICU, utilising machine learning-based mortality prediction models to aid ICU physicians in clinical practice is feasible. We plan to computerise a GBM-based tool for predicting the risk of in-hospital death amongst patients with sepsis in the future and integrate it into the existing ICU information system for real-time patient mortality monitoring and clinical decision support. Our future studies would prospectively evaluate the effectiveness of this mortality prediction model and system and check whether it improves the outcome of sepsis patients in clinical practice.

## Data Availability

The dataset used in this study, MIMIC III, is available at https://mimic.physionet.org/.

## Abbreviations

ICU: Intensive Care Unit
MIMIC: Medical Information Mart for Intensive Care
LASSO: Least Absolute Shrinkage and Selection Operator
GBM: Gradient Boosting Machine
RF: Random Forest
LR: Logistic Regression
AUROC: Area Under the Receiver Operating Characteristic curve
APACHE: Acute Physiology And Chronic Health Evaluation
SAPS: Simplified Acute Physiology Score
SOFA: Sequential Organ Failure Assessment
SSS: Sepsis Severity Score
BIDMC: Beth Israel Deaconess Medical Center
ICD-9-CM: International Classification of Diseases, ninth revision, Clinical Modification
